# Retraction: Resveratrol inhibits invasion and metastasis of colorectal cancer cells via MALAT1 mediated Wnt/β-catenin signal pathway

**DOI:** 10.1371/journal.pone.0328012

**Published:** 2025-07-14

**Authors:** The PLOS One Editors

After this article [1] was published, concerns were raised regarding Figs 2–8.

Specifically, there appears to be partial or full overlap between the following panels:

- The Invasion (48 h) - LoVo panel in Fig 2C and the Invasion (48 h) - over/MALAT1 panel in Fig 4B.
- The Invasion (48 h) - LoVo panel of Fig 4B, the Invasion (48 h) - Vector panel of Fig 4B and the Invasion (48 h) - over/MALAT1 panel in Fig 6A.
- The Migration (24 h) - LoVo panel in Fig 4B and the Migration (24 h) - over/MALAT1 panel in Fig 6A.
- The β-actin panels in Figs 3A and 3B.
- The β-actin panels in Figs 5A and 5B.
- Lanes 2–3 of the MMP-7 panel in Fig 5B and lanes 1–2 of the β-catenin (Nuclear) – shRNA/MALAT1 panel in Fig 7B.
- The Invasion (48 h) - vector and Invasion (48 h) - over/MALAT1 panels in Fig 8E, when flipped horizontally.
- The upper panels in Fig 7A in [1] and the lower panels of Fig 4E in [2] (retracted in [3]).
- The lower panels in Fig 7A in [1] and the upper panels of Fig 4E in [2] (retracted in [3]).

The first author stated that errors were made during the preparation of Figs 2C, 4B and 6A and provided what is stated to be the available original image and quantitative data underlying all figures in [1], and updated versions of Figs 4B and 6A. However, both the underlying image data and the updated figures provided raised further concerns about image integrity.

Regarding Figs 3 and 5, the first author stated that the same internal reference bands (β-actin) were presented in each figure because the original experiments were performed simultaneously with the same internal reference.

During editorial follow-up on another *PLOS One* article [4], the first author of [4] provided underlying images for Fig 2E of [4]. PLOS identified what appears to be partial and full panel overlap between that set of underlying data and panels in Figs 2C, 4B and 6A of [1].

Given the nature and extent of the above unresolved concerns which bring the reliability and integrity of the results and conclusions into question, the *PLOS One* Editors retract articles [1] and [4]. The Retraction notice for [4] can be found in [5].

QJ agreed with the retraction. XL, XF, LZhang, HS, LZhou, JS, JC, JQ, JR, and QL either did not respond directly or could not be reached.

## References

[1] Ji Q, Liu X, Fu X, Zhang L, Sui H, Zhou L, et al. Resveratrol inhibits invasion and metastasis of colorectal cancer cells via MALAT1 mediated Wnt/β-catenin signal pathway. PLoS One. 2013;8(11):e78700. doi: 10.1371/journal.pone.0078700 24244343 PMC3823921

[2] Chen F, Qi S, Zhang X, Wu J, Yang X, Wang R. lncRNA PLAC2 activated by H3K27 acetylation promotes cell proliferation and invasion via the activation of Wnt/β‑catenin pathway in oral squamous cell carcinoma. Int J Oncol. 2019;54(4):1183–94. doi: 10.3892/ijo.2019.4707 30720068 PMC6411352

[3] Chen F, Qi S, Zhang X, Wu J, Yang X, Wang R. [Retracted] lncRNA PLAC2 activated by H3K27 acetylation promotes cell proliferation and invasion via the activation of Wnt/β‑catenin pathway in oral squamous cell carcinoma. Int J Oncol. 2025;66(5):37. doi: 10.3892/ijo.2025.5743 40183411 PMC12068850

[4] Liu X, Ji Q, Ye N, Sui H, Zhou L, Zhu H, et al. Berberine Inhibits Invasion and Metastasis of Colorectal Cancer Cells via COX-2/PGE_2_ Mediated JAK2/STAT3 Signaling Pathway. PLoS One. 2015;10(5):e0123478. doi: 10.1371/journal.pone.0123478 25954974 PMC4425560

[5] The *PLOS One* Editors. Retraction: Berberine inhibits invasion and metastasis of colorectal cancer cells via COX-2/PGE_2_ mediated JAK2/STAT3 signaling pathway. PLoS ONE. 2025;20(7):e0328009. doi: 10.1371/journal.pone.0328009 PMC12258545 40658638

